# Emotional Intelligence, Instructor Warmth, and Metacognitive Effort Regulation in Nursing Students: An Exploratory Two-Institution Correlational Study in China and Pakistan

**DOI:** 10.3390/bs16101823

**Published:** 2026-10-05

**Authors:** Javed Iqbal, Zarqa Farooq Hashmi, Muhammad Zaheer Asghar, Muhammad Naseem Abid

**Affiliations:** 1 School of Artificial Intelligence for Humanities, Zhejiang International Studies University, Hangzhou 314400, China; javed@zisu.edu.cn; 2 School of Education, Huazhong University of Science and Technology, Wuhan 430000, China; naseemabid@ue.edu.pk; 3 Department of Education, University of Helsinki, 00014 Helsinki, Finland

**Keywords:** emotional intelligence, relational instructor warmth, metacognitive effort regulation, nursing students, structural equation modeling

## Abstract

Understanding how psychological attributes are associated with academic behavior is fundamental to educational psychology. This exploratory study investigated the relationships between emotional intelligence (EI), relational instructor warmth (RIW), and a newly developed measure of metacognitive effort regulation (MER) among nursing students in Pakistan and China. While prior research has examined these constructs separately, their interrelationships across different institutional contexts remain underexplored. A questionnaire survey containing 36 statements was completed by 581 nursing students in Pakistan and China, with responses collected on a 5-point Likert scale. The relationships were analyzed via partial least-squares structural equation modeling. The results indicate that emotional intelligence is directly and indirectly related to metacognitive effort regulation. Specifically, all four aspects of emotional intelligence (self-awareness, self-regulation, motivation, and social skills) were positively associated with metacognitive effort regulation. These associations are preliminary and require confirmation with validated measures. Relational instructor warmth was also positively correlated with metacognitive effort regulation, suggesting its potential association with higher metacognitive engagement and effort regulation in an academic context. Relational instructor warmth played a partial mediating role in the relationship between emotional intelligence and metacognitive effort regulation. Multigroup analysis revealed one significant difference between the two participating institutions: the path from self-regulation to relational instructor warmth was significantly stronger among students at the participating Pakistani institution than at the participating Chinese institution (*p* = 0.004). This institutional difference suggests that context-specific educational interventions may be more effective than one-size-fits-all approaches. These findings suggest that students with higher levels of emotional intelligence and relational instructor warmth may report better metacognitive regulation, which is associated with higher metacognitive effort regulation. As an exploratory study, the findings are preliminary and hypothesis-generating; they require confirmation using validated measures and longitudinal designs. The paper concludes by discussing the implications of these results and offers suggestions for future research.

## 1. Introduction

Nursing education presents unique challenges for students, including high levels of stress, academic pressure, and cognitive overload (da Silva, 2025). These challenges often result in emotional reactions, such as frustration, lack of motivation, disengagement, and metacognitive laziness, which can hinder students’ learning outcomes (Dubovi & Adler, 2024; Wang & Zheng, 2023). Emotional intelligence (EI) has been shown to be associated with better academic performance and improved well-being. Previous research highlights the role of EI in relation to cognitive outcomes (J. Iqbal et al., 2021a). However, the specific association of EI with metacognitive effort regulation (MER) through relational instructor warmth (RIW) among nursing students in Pakistan and China has not been fully explored.

The EI theory consists of components such as self-awareness (SA), self-regulation (SR), motivation (M), and social skills (SS) (Boyatzis et al., 2000; Goleman, 1998). These dimensions can help nursing students navigate the emotional and cognitive challenges they face during their academic journeys (J. Iqbal et al., 2022). However, the specific role of each adapted dimension of EI in relation to MER has not been fully explored, particularly in nursing education. Based on these assumptions, the present study explored the association of EI with RIW and MER, focusing on nursing students’ academic experiences.

MER involves overcoming cognitive avoidance, procrastination, and lack of engagement in reflective thinking (Fan et al., 2024). EI is associated with an environment in which students deeply engage in self-regulatory processes, with RIW characterized by supportive and empathetic interactions, which may encourage students to embrace cognitive challenges (J. Iqbal et al., 2022). Together, EI and RIW are associated with students’ M, emotional regulation, and critical thinking, which are linked to effective MER (Dewi et al., 2024; Fan et al., 2024; J. Iqbal et al., 2021a).

Relational instructor warmth refers to students’ perception that their instructor is caring, approachable, respectful, and supportive, and personally concerned about their academic and emotional experience in the learning environment (J. Iqbal et al., 2022), which is linked to students’ metacognitive processes and higher metacognitive effort regulation, possibly by providing a safe space where students feel valued and motivated to engage in reflective thinking (Fan et al., 2024). When teachers demonstrate warmth, students are more likely to report SR skills, effectively manage their learning, and remain committed to their academic goals. This nurturing relationship is associated with higher EI and greater involvement in learning, which is linked to students’ metacognitive processes and metacognitive effort regulation (Dewi et al., 2024; Fan et al., 2024; J. Iqbal et al., 2021a).

Most studies have suggested that the role of EI in learning requires further exploration (J. Iqbal et al., 2022). EI is associated with students’ skills necessary for MER (Dewi et al., 2024; Fan et al., 2024; J. Iqbal et al., 2021a). It has been argued that EI is a domain-specific construct, particularly in nursing education and other academic fields. Students with high EI are more confident in their ability to perform tasks within a specific domain while also recognizing their weaknesses in other areas (J. Iqbal et al., 2022). Therefore, this study examined the significant role of EI in relation to MER in nursing education. To the best of our knowledge, the literature suggests that the positive relationships between EI, RIW, and MER have not been sufficiently explored, particularly in the context of nursing education.

Given these gaps, there is a need for further investigation of the implications of EI, RIW, and MER in nursing learning environments. Therefore, this study aimed to explore how EI is associated with MER, with RIW serving as a mediating variable in this relationship. In this paper, we adopted the framework presented in EI theory (Boyatzis et al., 2000; Goleman, 1998). Metacognitive laziness theory explains how cognitive avoidance and procrastination interact in relation to individual metacognitive behaviors, especially in educational contexts (Fan et al., 2024). We also draw on social exchange theory (SET) (Cook et al., 2013), which is distinct from but complementary to social learning theory, to explore the role of social interactions in addressing learning challenges (J. Iqbal et al., 2022; Sulistyan, 2020). Despite the extensive literature on EI and metacognitive effort regulation, existing studies have proposed only partial models that, although statistically valid, do not fully capture the complexity of the relationships involved. Furthermore, the mediating role of RIW in EI and MER remains unclear (Ackerman et al., 2023; Balconi et al., 2023; Wirth et al., 2025). The core objective of this study was to explore the association of EI with MER in nursing education. This study also investigates the role of RIW in this relationship. To provide a comprehensive understanding of this phenomenon, this study examined several variables identified in the literature as related to the associations among EI, RIW, and MER. A quantitative approach was employed in this study, which involved 581 nursing students from Pakistan and China. Structural equation modeling was used to test the proposed hypotheses.

This study makes several key contributions to this field. First, it expands the literature on the association of EI with MER. Second, this study introduces a synthesized research model that explores the indirect association of EI with MER. Third, it provides empirical evidence and robust statistical analysis, clarifies both direct and indirect relationships, and illustrates how EI in conjunction with RIW is positively associated with MER among nursing students. Finally, this study highlights the importance of EI and demonstrates how RIW mediates its association with MER. Overall, these insights may be valuable for both practitioners and academics and offer a deeper understanding of these dynamics in nursing education. However, given the absence of a validated measure of metacognitive effort regulation in nursing education and the underexplored relationships among EI, RIW, and MER in cross-cultural contexts, this study is positioned as exploratory. Its primary aim is to examine whether a newly developed MER measure demonstrates adequate psychometric properties and whether theoretically expected relationships with EI and RIW are observed. The findings are intended to generate hypotheses for future confirmatory research rather than to provide definitive tests of causal relationships. This framework encourages further research to address the gaps identified and to guide the formulation of the following research questions:

***Research Question 1:** How is EI associated with MER among nursing students?*
***Research Question 2:** How is RIW associated with MER?*
***Research Question 3:** How does RIW mediate the relationship between EI and MER?*

The article begins with an introduction, research questions, and significance of the study, followed by a brief outline of the structure of the article. Section 2 critically examines previous studies and research models and develops hypotheses. Section 4 details the research methodology, including design, population, sampling techniques, data collection methods, instrumentation, and data analysis procedures. Section 8 presents the key findings through Tables, Figures and Analysis. Section 13, the discussion, concludes the paper, highlights implications and limitations, and offers directions for future research.

## 2. Literature Review and Theoretical Framework

### 2.1. Emotional Intelligence: Concept and Theory

EI comprises two core elements: emotion and intelligence. Emotions are feelings that individuals experience, which can stem from both internal and external circumstances. These emotional responses act as energy sources, providing valuable information about these conditions and motivating individuals to respond accordingly (Zhoc et al., 2018). Emotions are also seen as planned reactions to events that involve physiological, experiential, and cognitive components. On the other hand, intelligence refers to the cognitive abilities that enable individuals to think, learn, and solve problems effectively in various contexts (J. Iqbal et al., 2021a; Olson et al., 2019).

Zhang et al. (2020) used the term “EI,” which laid the foundation for the development of various EI theories and models. These models explore different facets of EI, including EI skills and traits, and mixed models. Ability-based EI models focus on psychological capabilities that help individuals process emotional information to achieve cognitive outcomes (J. Iqbal et al., 2021b). These models outline the cognitive abilities necessary to manage emotion-related issues. On the other hand, mixed models of EI blend these cognitive skills with personality traits such as optimism, enthusiasm, and self-confidence (Jan & Anwar, 2019).

In the present study, we utilized the trait model of EI, as conceptualized by J. Iqbal et al. (2021a). In addition, a three-stream model was applied to categorize EI (Ashkanasy & Daus, 2005). This model divides EI into three streams: Stream 1 focuses on the four-branch ability model, Stream 2 includes various self- and peer-report measures, and Stream 3 introduces aspects not covered by the ability model (Cabello et al., 2023). In this study, emphasis was placed on Stream 2, which relied on self-report measures, adapting the SA, SR, M, and SS dimensions with RIW and MER.

Goleman (1998) model conceptualizes EI as a multidimensional construct comprising four interrelated but distinct dimensions: self-awareness (SA), self-regulation (SR), motivation (M), and social skills (SS). Self-awareness involves recognizing and understanding one’s emotions and their association with cognition and behavior. Self-regulation refers to the capacity to manage and modulate emotional responses, particularly in challenging situations. Motivation encompasses intrinsic drive, persistence, and goal-directedness. Social skills involve the ability to build relationships, communicate effectively, and navigate social interactions. Critically, these dimensions operate synergistically: SA provides the foundation for SR, which in turn supports M and SS. This theoretical structure suggests that each dimension may be uniquely associated with learning outcomes while also operating through shared pathways. This study adopts Goleman’s four-dimension trait model as the theoretical basis for examining how EI is associated with learning behaviors.

### 2.2. Relational Instructor Warmth

Relational instructor warmth refers to the emotional connections and positive interactions between students and their instructors in an educational setting. It includes aspects such as social integration, belonging, and involvement that contribute to a supportive learning environment. RIW is associated with a sense of attachment to teachers and higher students’ confidence and M, particularly during emotional or cognitive challenges (Huang, 2021; J. Iqbal et al., 2021a; Olson et al., 2019). It is closely tied to emotional engagement, which extends beyond the classroom to interactions with academic advisors and faculty, associated with a sense of belonging and better learning outcomes (J. Iqbal et al., 2021a). In nursing education, RIW involves supportive and empathetic relationships that foster academic success and well-being. Relational instructor warmth is associated with metacognitive engagement, in which students manage their learning and address challenges, such as cognitive avoidance, procrastination, and a lack of engagement in reflective thinking. It is defined by how students perceive instructors as supportive, empathetic, encouraging, and emotionally engaged and is assessed through self-reports of interactions, supportiveness, and the classroom’s emotional climate (Dewi et al., 2024; Fan et al., 2024; J. Iqbal et al., 2021a).

Social Exchange Theory (Cook et al., 2013) provides a theoretical lens for understanding RIW. SET posits that social relationships are maintained through reciprocal exchanges of resources, including emotional support, respect, and engagement. In educational contexts, SET predicts that when students perceive their instructors as warm, supportive, and emotionally engaged, they feel obligated to reciprocate through increased engagement, effort, and participation (Sulistyan, 2020). This reciprocal dynamic is particularly relevant to understanding how RIW may mediate the relationship between students’ emotional competencies and their cognitive engagement. Students with higher EI may be more adept at eliciting and interpreting supportive responses from instructors, thereby initiating a positive exchange cycle that enhances metacognitive engagement.

### 2.3. Metacognitive Effort Regulation

Metacognitive effort regulation involves activating strategies such as self-regulation, goal setting, and self-monitoring to effectively manage learning processes (Fan et al., 2024). Effort regulation is vital for better academic performance by encouraging active learning, reflecting on progress, and overcoming issues such as procrastination and cognitive barriers. Effort regulation is defined as the extent to which students invest mental effort, persist with difficult tasks, and resist cognitive shortcuts, measured through self-reports on their learning management and task engagement. In this study, metacognitive effort regulation refers to current engagement in effortful cognitive strategies that counteract tendencies toward cognitive avoidance (Dewi et al., 2024; J. Iqbal et al., 2021a).

Fan et al. (2024) define metacognitive laziness as the tendency to avoid effortful cognitive processes, including procrastination, cognitive avoidance, and disengagement from reflective thinking. However, overcoming metacognitive laziness requires active metacognitive effort regulation and engagement in deeper cognitive strategies. The capacity to regulate effort depends on both individual emotional resources (e.g., EI) and contextual support (e.g., RIW), making this construct particularly relevant for understanding how emotional and relational factors interact to shape learning behaviors. To anchor metacognitive effort regulation theoretically, we draw on effort discounting theory (Kool & Botvinick, 2014) and cognitive avoidance theory (Borkovec et al., 2004).

Effort discounting theory posits that individuals devalue cognitive rewards as a function of the effort required to obtain them. Cognitive avoidance theory frames laziness as avoidance of aversive cognitive content. Together, these theories suggest that effort regulation requires overcoming both effort discounting and cognitive avoidance. In the present study, metacognitive effort regulation is conceptualized as the ongoing engagement in effortful cognitive strategies that counteract tendencies toward cognitive avoidance. This conceptualization distinguishes effort regulation from EI as an emotional competency and from general self-regulation, which concerns emotional and behavioral control rather than effort-related cognitive choices in learning tasks.

Fan et al. (2024) examined metacognitive laziness in an AI-supported learning context and did not validate a general effort regulation scale for nursing students. The five-item effort regulation measure used here was therefore adapted and newly developed for this research; development and expert review are reported in Section 5. This measure should be considered exploratory, and its psychometric properties require independent validation before the findings can be treated as confirmatory. This conceptualization aligns with prior work on cognitive effort regulation and self-regulated learning (Pintrich, 2000; Zimmerman, 2000).

### 2.4. Emotional Intelligence Studies in Pakistan and China

Studies on EI in developing and emerging countries, especially Pakistan and China, have been relatively limited, although some have underscored its significant association with the academic performance of undergraduate students (J. Iqbal et al., 2022, 2021a). For example, Xu et al. (2021) reported a moderate positive correlation between emotional intelligence and subjective well-being among Chinese samples, with variations influenced by the type of EI measurement, age, and employment status of participants. Ghafoor et al. (2019) explored the interplay between emotional intelligence, metacognition, and negative coping strategies on health-related quality of life in a cross-cultural study of German and Pakistani individuals, revealing distinct cultural variations in the associations of these psychological constructs. These insights highlight the importance of EI for nursing students in Pakistan and China, particularly in helping them cope with the challenges of cognitive avoidance, with the support of RIW.

### 2.5. Research Model

**Figure 1:**
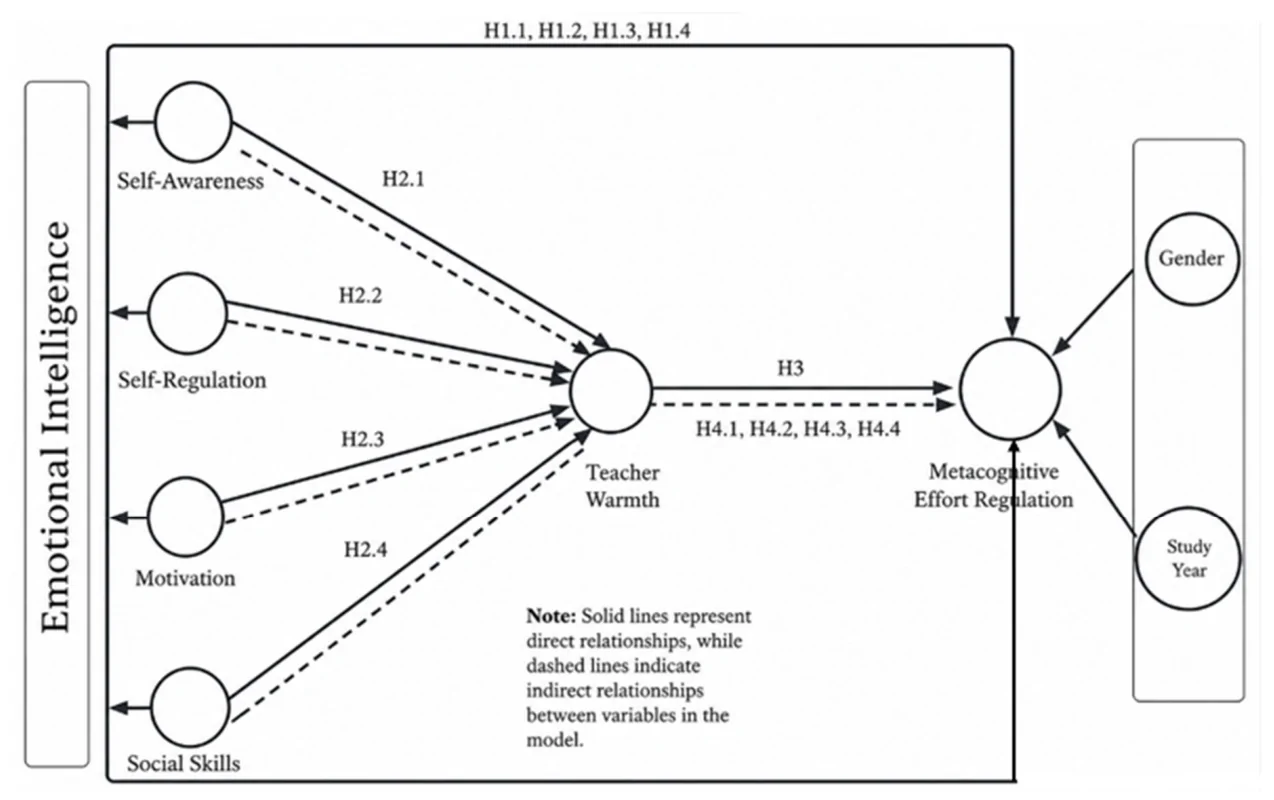
Research model: A path diagram showing self-awareness, self-regulation, motivation, and social skills as independent variables leading to metacognitive effort regulation as the dependent variable, with relational instructor warmth as a mediating variable between emotional intelligence dimensions and MER.

The research model was designed by adapting the EI theory proposed by Boyatzis et al. (2000); (Goleman, 1998), SET, and a literature review (J. Iqbal et al., 2022; Sulistyan, 2020) to explore the association of EI with MER through RIW. In this study, EI is conceptualized as a multidimensional construct comprising four distinct dimensions: self-awareness (SA), self-regulation (SR), motivation (M), and social skills (SS). EI was modeled as four separate first-order constructs rather than as a second-order latent construct for three reasons. First, Goleman (1998) theory posits that the four dimensions are distinct competencies that may have differential relationships with outcomes. Second, the study’s hypotheses specify differential relationships for each dimension. Third, a second-order model would obscure these differential relationships. However, we acknowledge that a second-order model is a viable alternative specification. Future research should compare model fit between first-order and second-order specifications. Previous research has shown that EI and RIW have significant associations with students’ learning behaviors. EI has been shown to be associated with cognitive function and with students’ cognitive engagement and academic outcomes (J. Iqbal et al., 2022, 2021a). The current study hypothesizes that RIW, associated with EI, mediates the relationship between EI (SA, SR, M, and SS) and MER. This study examined how EI is associated with MER through RIW. High-EI students foster a reciprocal warm environment that is associated with higher RIW (Dewi et al., 2024; Fan et al., 2024; J. Iqbal et al., 2021a). This study clarifies how EI (SA, SR, M, and SS) is associated with RIW for MER, suggesting that higher EI and RIW may be linked to higher MER, as shown in the research model (see Figure 1).

## 3. Hypothesis Development

### 3.1. Emotional Intelligence and Metacognitive Effort Regulation

The relationship between EI and MER can be theoretically explained through the mechanisms proposed by Goleman (1998) and extended by recent empirical work (J. Iqbal et al., 2022, 2021a). Each EI dimension is uniquely associated with the cognitive and emotional processes involved in regulating effort.

**Self-Awareness and MER (H1.1).** Self-awareness enables students to recognize when their emotional states, such as anxiety, boredom, or frustration, are interfering with their cognitive engagement. This recognition is a prerequisite for taking corrective action. Students with high SA can identify when they are engaging in cognitive avoidance or procrastination and consciously adjust their learning strategies (J. Iqbal et al., 2021b). In nursing education, where students face high-stress clinical environments and complex academic demands, SA may be particularly important for recognizing when emotional fatigue leads to superficial learning. Theoretically, SA is associated with MER, possibly through metacognitive monitoring necessary to detect and address low-effort thinking patterns. Therefore:

**H1.1.**  *Self-awareness is positively associated with metacognitive effort regulation*.

**Self-Regulation and MER (H1.2).** Self-regulation involves the capacity to manage emotional responses and maintain goal-directed behavior despite distractions or difficulties. Students with high SR can inhibit impulsive tendencies toward cognitive avoidance and persist in effortful cognitive tasks (J. Iqbal et al., 2022). In the context of MER, SR is associated with students’ ability to resist the temptation to take mental shortcuts and instead engage in deeper, more effortful processing. This aligns with the broader literature on self-regulated learning, which demonstrates that effective emotional regulation supports cognitive regulation (Zhoc et al., 2018). For nursing students, SR may be particularly critical given the emotionally demanding nature of their training. Therefore:

**H1.2.**  *Self-regulation is positively associated with metacognitive effort regulation*.

**Motivation and MER (H1.3).** Motivation, as conceptualized within EI theory, refers to intrinsic drive, persistence, and the ability to delay gratification in pursuit of long-term goals. Students with high M are more likely to invest sustained effort in learning tasks, even when immediate rewards are absent (Boyatzis et al., 2000). This intrinsic drive is associated with higher metacognitive effort regulation, which is characterized by a preference for sustained effort over immediate cognitive ease. In nursing education, where students must master complex material and develop clinical reasoning skills, motivation may be essential for maintaining the cognitive effort required for MER. Therefore:

**H1.3.**  *Motivation is positively associated with metacognitive effort regulation*.

**Social Skills and MER (H1.4).** Social skills enable students to build supportive relationships with peers and instructors, creating a social context that encourages engagement and discourages cognitive avoidance. Students with strong SS are more likely to seek help when struggling, participate in collaborative learning, and benefit from the cognitive stimulation provided by social interaction (J. Iqbal et al., 2021a). These social resources may be indirectly associated with MER through accountability and providing external motivation. Additionally, social interaction itself can stimulate deeper cognitive processing, as students articulate and defend their ideas. Therefore:

**H1.4.**  *Social skills are positively associated with metacognitive effort regulation*.

### 3.2. Emotional Intelligence and Relational Instructor Warmth

The relationship between EI and RIW can be explained through both intrapersonal and interpersonal mechanisms. Intrapersonally, students with higher EI possess the emotional competencies that are associated with positive social interactions. Interpersonally, SET predicts that students who demonstrate emotional competence may elicit more supportive responses from instructors.

**Self-Awareness and RIW (H2.1).** Students with high SA are attuned to their own emotional states and can communicate their needs more effectively to instructors. This emotional clarity may be associated with more authentic and open relationships with teachers (Mastrokoukou et al., 2025). When students understand their emotional reactions to academic challenges, they may be more likely to seek appropriate support rather than withdrawing or acting out. Instructors may perceive such students as more receptive to support, thereby strengthening the relational bond. Therefore:

**H2.1.**  *Self-awareness is positively associated with relational instructor warmth*.

**Self-Regulation and RIW (H2.2).** Self-regulation enables students to manage negative emotions, such as frustration or anxiety, in ways that do not disrupt classroom relationships. Students who can regulate their emotional responses are more likely to interact with instructors in respectful and constructive ways, even when facing academic difficulties (J. Iqbal et al., 2021a). This emotional stability may lead instructors to perceive such students as more approachable and may encourage instructors to invest more emotional energy in the relationship. Therefore:

**H2.2.**  *Self-regulation is positively associated with relational instructor warmth*.

**Motivation and RIW (H2.3).** Motivated students demonstrate enthusiasm, persistence, and a positive attitude toward learning, which may elicit more positive responses from instructors. According to SET, instructors may reciprocate students’ demonstrated engagement with increased warmth and support (Sulistyan, 2020). Additionally, motivated students may seek out more frequent and meaningful interactions with instructors, thereby building stronger relationships. Therefore:

**H2.3.**  *Motivation is positively associated with relational instructor warmth*.

**Social Skills and RIW (H2.4).** Social skills are associated with the development of positive relationships with instructors. Students with strong SS can communicate effectively, express appreciation, and navigate the social dynamics of the classroom in ways that build rapport (J. Iqbal et al., 2022). These skills are particularly important for establishing the kind of warm, supportive relationship that characterizes RIW. Therefore:

**H2.4.**  *Social skills are positively associated with relational instructor warmth*.

### 3.3. Relational Instructor Warmth and Metacognitive Effort Regulation

The relationship between RIW and MER can be explained through multiple mechanisms grounded in SET and self-determination theory (Ryan & Deci, 2000). First, RIW is associated with a psychologically safe environment in which students feel comfortable taking intellectual risks and acknowledging difficulties without fear of judgment (Kong et al., 2025). This safety may be associated with lower anxiety that often triggers cognitive avoidance. Second, warm instructors offer emotional support that is associated with students’ persistence through challenging cognitive tasks (Li, 2025). Third, RIW may be associated with students’ sense of accountability; students who feel that their instructor cares about their learning may be more motivated to engage deeply rather than taking shortcuts. Finally, warm instructors may model engaged, reflective thinking, providing students with examples of effective metacognitive strategies. Therefore:

**H3.**  *Relational instructor warmth is positively associated with metacognitive effort regulation*.

### 3.4. The Mediating Role of Relational Instructor Warmth

The hypothesis that RIW mediates the relationship between EI dimensions and MER is grounded in the proposition that individual emotional competencies are expressed and amplified within supportive relational contexts. This argument integrates EI theory with SET.

According to EI theory, students with high EI possess the intrapersonal resources necessary for effective cognitive engagement. However, the expression of these resources may depend on the interpersonal context. SET predicts that when students perceive their instructors as warm and supportive, they experience a sense of reciprocal obligation that motivates them to invest in the relationship through increased engagement (Sulistyan, 2020). Thus, RIW may serve as a mechanism through which EI is associated with MER.

Specifically, students with high SA, SR, M, and SS may be more adept at developing warm relationships with instructors. These relationships, in turn, provide emotional support, encouragement, and accountability that are associated with MER. This mediation model suggests that the association of EI with MER is not solely direct but operates, in part, through the quality of the student-instructor relationship.

**H4.1.**  *Self-awareness is positively associated with MER through relational instructor warmth*.

**H4.2.**  *Self-regulation is positively associated with MER through relational instructor warmth*.

**H4.3.**  *Motivation is positively associated with MER through relational instructor warmth*.

**H4.4.**  *Social skills are positively associated with MER through relational instructor warmth*.

### 3.5. Theoretical Justification for Direction

The pathway EI to RIW to MER is theoretically grounded in the assumption that intrapersonal competencies precede interpersonal perceptions, which in turn shape cognitive-behavioral outcomes. EI is conceptualized as a relatively stable trait (Goleman, 1998); it resides within the individual and logically precedes relational perceptions (RIW). SET (Cook et al., 2013) then predicts that perceived warmth elicits reciprocal engagement, which is associated with MER. Reverse directions are less plausible: RIW to EI would require a single relationship to alter a stable trait; EI to MER to RIW would reverse the logical flow from perception to behavior; MER to EI has no theoretical basis. The cross-sectional design cannot definitively establish causality; the hypothesized direction represents the theoretically most plausible pathway.

## 4. Design and Sampling

To test these hypotheses, data were collected from students enrolled in nursing programs at one institution in Pakistan and one institution in China, using a convenience sampling technique. Convenience sampling was employed for practical reasons, as it allowed efficient data collection from accessible nursing student populations in both countries. However, this approach has limitations: it may introduce selection bias and does not guarantee representativeness of the broader nursing student population in either country. The findings should therefore be interpreted with caution, and the limitations of this sampling strategy are further discussed in Section 13. The data collection process began with formal approval from the School Ethical Research Committee of the Host University, following the guidelines of the Declaration of Helsinki. Before the study, comprehensive information about the research was given to students, and their consent and agreement were gained. Once approved, the research team coordinated with the instructors to facilitate data collection in their classes. The participants were thoroughly briefed on the study objectives, and informed consent was obtained to ensure voluntary participation and confidentiality of their responses.

The survey questionnaire was administered in printed format via in-person visits. A total of 700 students, recruited from one nursing college in Hubei, Wuhan (China) and one nursing college in Punjab, Lahore (Pakistan), were invited to participate. Of these, 589 completed questionnaires were returned, representing an 84.14% response rate. Participants were selected based on the following criteria: (1) enrollment as a nursing undergraduate student in the specified colleges; (2) age ≥ 18 years; and (3) voluntary participation with informed consent.

Participant flow and exclusions. Of the 700 distributed questionnaires, 589 were returned complete. Eight cases were identified as outliers using Z scores (threshold ±3.29) and were excluded from analysis, resulting in a final analytical sample of 581. All demographic, measurement, structural, and multigroup analyses reported in this manuscript are based on this final sample of 581.

**Figure 2:**
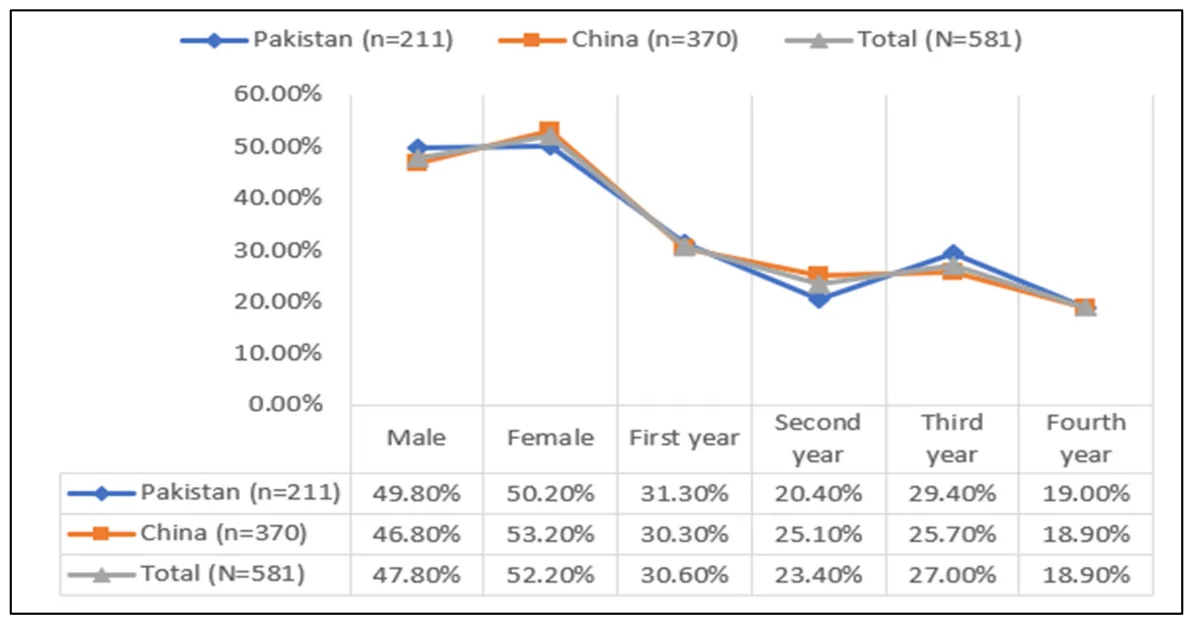
Demographic Distribution by Institution. Comparison of gender and year-of-study distributions across Pakistani (n = 211) and Chinese (n = 370) nursing student samples. Percentages are calculated within each group. The two samples show broadly comparable demographic profiles. Data are based on the final analytical sample (N = 581) after excluding 8 outliers.

Demographic characteristics for the final analytical sample (*N* = 581) are reported separately by institution in Figure 2. In Pakistan (n = 211), 49.8% of respondents were male and 50.2% were female; in China (*n* = 370), 46.8% were male, and 53.2% were female. Regarding year of study, the Pakistani sample comprised 31.3% first-year, 20.4% second-year, 29.4% third-year, and 19.0% fourth-year students. The Chinese sample comprised 30.3% first-year, 25.1% second-year, 25.7% third-year, and 18.9% fourth-year students. Across the full sample (*N* = 581), 47.8% were male, and 52.2% were female; 30.6% were first-year, 23.4% second-year, 27.0% third-year, and 18.9% fourth-year students. Year of study was included as a control variable because academic seniority may be associated with metacognitive skill development.

## 5. Questionnaire Design

In this study, EI served as the independent variable, while MER was the dependent variable. RIW was examined as a mediator of the relationship between EI and MER. A 36-item questionnaire, consisting of three sections, was developed to assess these variables. The questionnaire incorporated items from established, reliable, and valid constructs used in previous studies J. Iqbal et al. (2021a), and some new items were added to make it more reliable, valid, and contextual for this study. Five experts (two nursing education researchers, one educational psychologist, one psychometrician, one cross-cultural researcher) reviewed the questionnaire for content relevance, clarity, and conceptual distinctness. Based on their feedback, problematic items were reworded for clarity, and two items were revised to reduce overlap with other constructs. The revised instrument was then pilot-tested. Section 1 provided information about the study’s purpose, privacy, and anonymity, along with instructions for the respondents. Section 2 collected demographic data including sex, year of study, and country. Section 3 contained items related to EI (22 items), RIW (9 items), and MER (5 items), measured on a 5-point Likert-type scale ranging from 1 (strongly disagree) to 5 (strongly agree). As noted in Section 2.3, the MER scale was newly developed for this exploratory study; while the pilot study and expert review provide preliminary evidence of reliability and content validity, formal validation with independent samples was beyond the scope of this study.

### 5.1. Pilot Study

A pilot study was conducted with 50 nursing students (25 from the Pakistani institution, 25 from the Chinese institution). The pilot sample was selected to match the final sample on gender, year of study, and age. The pilot served three purposes: (a) to assess item clarity and comprehension, (b) to compute preliminary reliability coefficients, and (c) to identify problematic items.

**Item-level statistics.** Corrected item-total correlations ranged from 0.42 to 0.78. No item had a corrected item-total correlation below 0.30. Cronbach’s alpha coefficients were: SA = 0.87, SR = 0.89, M = 0.85, SS = 0.91, RIW = 0.92, MER = 0.86. Alpha-if-item-deleted values did not exceed the overall alpha for any construct, indicating that no item unduly suppressed reliability.

**Revisions based on pilot.** Two items were revised to reduce conceptual overlap with other constructs: SR2 and RIW1 (see Appendix A for revised wording). Minor wording changes were made to three additional items to improve clarity.

### 5.2. Expert Review

Five experts (two nursing education researchers, one educational psychologist, one psychometrician, one cross-cultural researcher) reviewed the questionnaire for content relevance, clarity, and conceptual distinctness. Experts were selected based on three criteria: (a) a doctoral degree in a relevant field, (b) at least five years of research or teaching experience in higher education, and (c) familiarity with survey instrument development.

Each expert received the full questionnaire with instructions to evaluate (a) whether each item was relevant to its intended construct, (b) whether each item was clearly worded, and (c) whether any items overlapped conceptually with items from other constructs. Experts provided written comments and suggested revisions.

Experts identified four items with conceptual overlap (SR2, SR3, RIW1, SS1). SR2 and RIW1 were revised to remove outcome language. SR3 and SS1 were retained because the overlap was judged minor and the items were considered essential for content coverage. Experts also suggested minor wording changes to three additional items (SR5, SS4, MER2) to improve clarity. Formal content validity indices (I-CVI, S-CVI) were not computed; this is acknowledged as a limitation and is consistent with the exploratory nature of the instrument development.

### 5.3. Translation and Administration

The instrument was developed in English. The Pakistani institution uses English as the medium of instruction; therefore, Pakistani participants completed the English version only. The Chinese institution uses Chinese as the medium of instruction; therefore, Chinese participants completed the Chinese version only.

The Chinese version was developed through a standard forward-back translation procedure: (a) forward translation by two bilingual researchers (Chinese-English), (b) back-translation by an independent translator who was blind to the original English version, (c) reconciliation by a three-member committee (two bilingual researchers and one methodologist), and (d) cognitive interviews with five Chinese nursing students to assess comprehension and cultural relevance. Minor wording adjustments were made based on cognitive interview feedback.

An Urdu version was not considered necessary because English is the medium of instruction at the Pakistani institution and all nursing students are proficient in English. The Pakistani institution’s language policy requires English proficiency for admission.

Semantic equivalence was established through the translation procedure described above and through MICOM analysis (see Section 8.2). MICOM establishes statistical comparability but does not establish linguistic equivalence; this is acknowledged as a limitation.

## 6. Measures

Emotional intelligence was measured using 22 items adapted from J. Iqbal et al. (2022), designed to capture four distinct dimensions: self-awareness (5 items), self-regulation (6 items), motivation (4 items), and social skills (7 items). Each dimension was treated as a separate construct in the analysis, consistent with Goleman (1998) theoretical framework and the study’s hypothesis structure.

*Self-Awareness:* The items measuring SA were developed and adapted from the work of J. Iqbal et al. (2021a) and comprised a total of five items. An example item is “I can recognize my emotions when faced with academic challenges.” The Cronbach’s alpha was 0.878, indicating that the scale was reliable (see Table 1).

*Self-regulation:* The items measuring SR were developed and adapted from the work of J. Iqbal et al. (2021a) and comprised a total of six items. An example item is, “I manage my emotions effectively when facing difficult learning tasks.” The Cronbach’s alpha was 0.902, indicating that the scale was reliable (see Table 1).

*Motivation:* The items measuring M were developed and adapted from the work of J. Iqbal et al. (2021a) and comprised a total of four items. An example item is: I take responsibility for my emotional reactions to academic challenges.” The Cronbach’s alpha was 0.863, indicating that the scale was reliable (see Table 1).

*Social Skills:* The items measuring SS were developed and adapted from the work of J. Iqbal et al. (2021a), comprising a total of seven items. An example item is, “I can adapt my communication style to suit different people in academic settings.” Cronbach’s alpha was 0.915, indicating that the scale was reliable (see Table 1).

*Relational instructor warmth:* The items measuring RIW were developed based on the study by J. Iqbal et al. (2021a) and included a total of nine items. An example item is, “My teacher shows genuine interest in my emotional well-being.” The Cronbach’s alpha was 0.934, indicating that the scale was reliable (see Table 1).

*Metacognitive effort regulation:* The five-item MER scale was newly developed for this study; Fan et al. (2024) provided the conceptual definition but did not validate a general MER scale for nursing students. “An example item is, ‘I use effortful cognitive strategies when completing academic tasks.’” The Cronbach’s alpha was 0.885, indicating that the scale was reliable (see Table 1).

## 7. Data Analysis Procedure

We employed rigorous analytical methods to confirm the validity, reliability, and credibility of our results using two efficient statistical software tools, SPSS and SmartPLS. Descriptive statistics for participant demographics were generated using SPSS 22, whereas SmartPLS 4 was used for both measurement and structural modeling. First, we analyzed the demographic data of the participants using descriptive statistics. The measurement model analysis involved assessing the factor loadings, Cronbach’s alpha, composite reliability (rho_a and rho_c), and convergent and discriminant validity. We addressed collinearity issues to improve the model fit and discussed the explanatory power of the model in detail. Finally, we applied structural equation modeling (SEM) to explore both direct and indirect relationships between the constructs in our model (Hair et al., 2019). Ensuring the accuracy of the data involved several steps. Although the consent form stated participants could skip questions, incomplete questionnaires were excluded from analysis. All 589 questionnaires returned were complete; no missing values were observed. Outliers were screened using Z scores (threshold ±3.29); 8 cases were identified and excluded, resulting in the final analytical sample of 581. Normality was checked by examining skewness and kurtosis, ensuring that they remained within the ±3 range. Common method bias was assessed using procedural techniques (anonymity, confidentiality assurances), Harman’s single-factor test, and a full collinearity VIF assessment. An unrotated principal component analysis of all 36 items extracted six components with eigenvalues greater than 1, cumulatively explaining 67.53% of the variance. The first component explained 28.61% of the variance, which is below the 50% threshold commonly used to indicate a dominant single factor. All inner VIF values ranged from 1.008 to 1.215, well below the threshold of 5. These results indicate that no substantial indication of common method bias was detected. However, these are screening procedures and do not definitively rule out bias (Hair et al., 2019; Q. Iqbal & Piwowar-Sulej, 2022).

## 8. Results

### 8.1. Measurement Analysis

To evaluate the reliability and validity of the constructs, key threshold values were applied to the indicators. Reliability was assessed using Cronbach’s alpha and composite reliability, with values exceeding the commonly accepted threshold of 0.70 indicating strong internal consistency. Convergent validity was examined through the average variance extracted, with values above 0.50, demonstrating that the constructs captured sufficient variance from their items. Discriminant validity was confirmed via the heterotrait–monotrait (HTMT) ratio, ensuring that each construct was distinct from the others, with threshold values less than 0.85. Item loadings exceeding 0.60 were considered indicative of an adequate representation of their respective constructs. Additionally, multicollinearity was checked by verifying that the variance inflation factors remained below the threshold of five, ensuring that there was no significant overlap between items. These criteria collectively establish the robustness of the measurement model used in the analysis (Hair et al., 2019).

Table 1 indicates that the constructs used in this study exhibited good reliability and validity. The scales for SA, SR, M, SS, RIW, and MER showed strong internal consistency, ensuring that the items within each construct reliably measured the intended factors. Item loadings were generally high, suggesting that each item effectively represented its respective construct. Additionally, multicollinearity analysis revealed no significant issues across the constructs, as the relationships between the items were within acceptable limits. This suggests that the constructs are distinct from one another and do not overlap, thus enhancing the overall quality of the measurement model. The results indicate that the measures used are robust, with strong reliability and validity and no substantial concerns about multicollinearity.

The heterotrait-monotrait ratio (HTMT) was used to assess discriminant validity. All HTMT values were below the recommended threshold of 0.85 (Henseler et al., 2015), confirming that each construct is empirically distinct. The highest HTMT value was between metacognitive effort regulation and relational instructor warmth (0.473), followed by metacognitive effort regulation and motivation (0.460), metacognitive effort regulation and social skills (0.449), metacognitive effort regulation and self-regulation (0.440), and metacognitive effort regulation and self-awareness (0.410). All other HTMT values ranged from 0.014 to 0.325. Notably, the HTMT value between self-regulation and metacognitive effort regulation (0.440) was below the 0.85 threshold, suggesting that these two regulation-related constructs may be empirically distinguishable. However, this value also indicates some conceptual overlap that warrants further investigation in future validation studies. As an exploratory study, these results should be interpreted as tentative.

The R-squared values indicated that the model explained 41.1% of the variance in MER (R^2^ = 0.411, adjusted R^2^ = 0.404) and 17.0% of the variance in RIW (R^2^ = 0.170, adjusted R^2^ = 0.165). By PLS-SEM benchmarks (Hair et al., 2019), these values represent weak-to-moderate and weak explanatory power, respectively. The F-squared effect sizes were small by Hair et al.’s (2019) benchmarks (0.02 = small, 0.15 = medium, 0.35 = large), with social skills showing the strongest effect (F^2^ = 0.073), followed by RIW (F^2^ = 0.066), SA (F^2^ = 0.055), M (F^2^ = 0.054), and SR (F^2^ = 0.051). Gender and study years had negligible effects (F^2^ = 0.001 each). For RIW, self-regulation demonstrated the strongest effect (F^2^ = 0.045), followed by social skills (F^2^ = 0.031) and motivation (F^2^ = 0.022), with self-awareness showing a negligible effect (F^2^ = 0.012). Model fit indices indicated good model fit, as the SRMR values for the saturated and estimated models were 0.035 and 0.036, respectively (below the recommended threshold of 0.08), and the NFI values were 0.923 and 0.922, respectively (exceeding the recommended threshold of 0.90), suggesting that the proposed model is well-specified (Hair et al., 2019).

### 8.2. Direct Relationship

**Figure 3:**
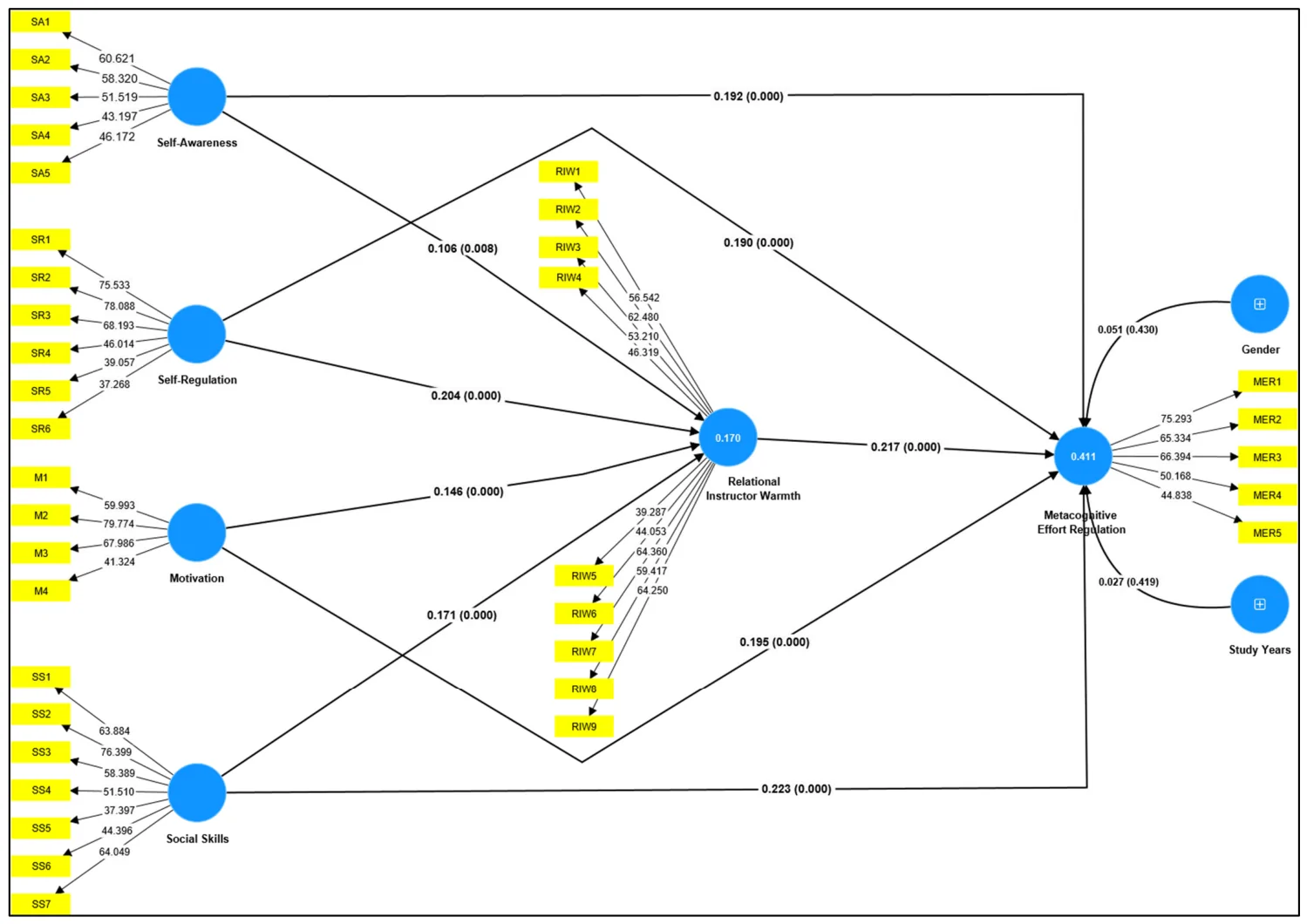
*Structural Model with Path Coefficients.* **Note.** SA = self-awareness; SR = self-regulation; M = motivation; SS = social skills; RIW = relational instructor warmth; MER = metacognitive effort regulation. EI was modeled as four separate first-order dimensions (SA, SR, M, SS) rather than as a single overall construct. Each dimension was entered as a separate predictor in the structural model. Solid lines indicate significant paths (*p* < 0.05).

The results indicated that self-awareness had a positive direct relationship with MER (β = 0.192, *p* < 0.001), supporting hypothesis H1.1. Self-regulation also had a positive and significant direct relationship with MER (β = 0.190, *p* < 0.001), supporting hypothesis H1.2. Motivation had a positive direct relationship with MER (β = 0.195, *p* < 0.001), supporting hypothesis H1.3. Social skills had a significant positive relationship with MER (β = 0.223, *p* < 0.001), supporting hypothesis H1.4 (see Table 2 and Figure 3).

The relationships between emotional intelligence constructs and relational instructor warmth were all significant and positive. Results indicated that self-awareness (β = 0.106, *p* = 0.008), self-regulation (β = 0.204, *p* < 0.001), motivation (β = 0.146, *p* < 0.001), and social skills (β = 0.171, *p* < 0.001) each showed a significant positive relationship with relational instructor warmth, supporting hypotheses H2.1, H2.2, H2.3, and H2.4 (see Table 2 and Figure 3).

Finally, results indicated that relational instructor warmth had a significant positive direct effect on MER (β = 0.217, *p* < 0.001), supporting hypothesis H3. With respect to the control variables, neither gender (β = 0.051, *p* = 0.430) nor study years (β = 0.027, *p* = 0.419) was significantly associated with MER, indicating that no statistically significant association was detected between these demographic factors and the outcome variable in the present sample (see Table 2 and Figure 3). A robustness check was conducted by re-estimating the model without control variables. The substantive conclusions were unchanged: all hypothesized paths remained significant, and the pattern of results was identical. The control variables were therefore retained in the reported model for completeness.

Table 3 presents the results of the indirect relationships in the structural equation model, examining the associations of emotional intelligence constructs with MER through relational instructor warmth. The results indicated that self-awareness (β = 0.023, *p* = 0.015), self-regulation (β = 0.044, *p* < 0.001), social skills (β = 0.037, *p* < 0.001), and motivation (β = 0.032, *p* = 0.001) each had positive and significant indirect relationships with MER through relational instructor warmth, supporting hypotheses H4.1, H4.2, H4.3, and H4.4. These findings indicate that relational instructor warmth mediates the relationship between emotional intelligence constructs and metacognitive effort regulation (see Table 3 and Figure 3). The indirect effects were statistically significant but small (β = 0.023 to 0.044). These small coefficients suggest that RIW may have limited practical mediating importance, although the consistency of the pattern across all four EI dimensions supports the theoretical model.

***Type of mediation:*** Partial mediation was observed for all four EI dimensions. As shown in Table 2, each EI dimension had a significant direct effect on MER (all *p* < 0.001). As shown in Table 3, each EI dimension also had a significant indirect effect on MER through RIW, with bootstrapped 95% confidence intervals not containing zero. Total effects (direct + indirect) are reported in Table 4. Because both direct and indirect effects were significant, RIW partially mediates the relationship between each EI dimension and MER. This indicates that EI is associated with MER both directly and indirectly through relational instructor warmth. The significance of each indirect pathway was confirmed by bootstrapped 95% confidence intervals that did not contain zero (Table 3).

To test cross-institutional differences, we used Henseler’s PLS-MGA (Henseler et al., 2016a) The SmartPLS 4 ‘PLS-MGA’ option was used, which implements a one-tailed test based on bootstrap differences. Significance was assessed at *p* < 0.05 (one-tailed). MICOM establishes statistical comparability but does not establish linguistic or semantic equivalence; translation procedures are described in Section 5. As shown in Table 5, measurement invariance was assessed using MICOM. Step 2 (compositional invariance) was established for all constructs (all *p* > 0.05), confirming that the constructs have the same meaning across country groups. Step 3a (equal means) was also established for all constructs (all *p* > 0.05). However, Step 3b (equal variances) revealed significant differences for motivation (*p* = 0.002) and self-awareness (*p* = 0.014), indicating partial measurement invariance. These variance differences suggest that students at the two participating institutions may differ in the dispersion of motivation and self-awareness scores, though the constructs remain conceptually comparable. As compositional invariance (Step 2) was established, partial measurement invariance is sufficient to proceed with multigroup analysis (Henseler et al., 2016a). Table 6 presents the country-specific path coefficients and the multigroup analysis results. The only significant group difference was found for the path from self-regulation to relational instructor warmth (difference = 0.231, *p* = 0.004), which was significantly stronger among students at the participating Pakistani institution (β = 0.348) than among students at the participating Chinese institution (β = 0.116). All other direct and indirect path differences were non-significant (all *p* > 0.05).

## 9. Discussion

This exploratory study examined the correlational relationships between EI and MER through RIW among nursing students in Pakistan and China. The theoretical model developed in this study was tested and refined based on the results of the analysis. As an exploratory study, the primary contribution of this research is to identify patterns and generate hypotheses rather than to provide definitive tests of relationships. The findings suggest that EI dimensions and RIW are associated with MER, but these relationships require confirmation using validated measures and longitudinal designs. Most previous studies were conducted in developed countries, with limited research in emerging nations such as Pakistan and China (J. Iqbal et al., 2022, 2021a). Additionally, there is a lack of research on the role of higher education in enhancing MER among students in China and Pakistan. To the best of our knowledge, this study is one of the first to investigate the correlational associations between EI and MER at the participating institutions in Pakistan and China, particularly with RIW as a mediator.

Social skills showed the largest standardized coefficient among the EI dimensions, followed by motivation, self-awareness, and self-regulation. However, the differences among these coefficients were small, suggesting that all four EI dimensions were comparably associated with MER, consistent with Goleman (1998) synergistic view. RIW showed a comparable association with MER, indicating that relational support and individual competencies were similarly associated with the outcome. The most notable preliminary finding was the stronger SR to RIW association among students at the participating Pakistani institution than at the participating Chinese institution. However, this difference is limited to the two participating institutions and cannot be attributed to national culture. It may reflect institutional, curricular, regional, or sociodemographic characteristics not measured in this study. It is also important to note that Hofstede’s (2001) framework places China higher than Pakistan on power distance, which contradicts a simple cultural explanation. These interpretations are speculative and warrant future research that directly measures cultural values and institutional characteristics across multiple institutions.

The study examined the direct correlational relationships between EI and MER and found that all four EI dimensions (SA, SR, M, and SS) had significant positive associations with MER. These patterns are consistent with hypotheses H1.1 through H1.4 and warrant further confirmatory testing. These findings align with the notion that EI dimensions are positively linked to cognitive improvements among university students (J. Iqbal et al., 2021a). Moussa et al. (2024) reported a positive connection between EI and cognitive function among Lebanese health care professionals. Our results showed that both SA and M positively correlated with MER. This suggests that nursing students’ self-awareness of their emotional states and their intrinsic motivation are associated with higher metacognitive effort regulation (J. Iqbal et al., 2022).

This study explored the positive and direct correlational relationships of EI with RIW, highlighting that EI (SA, SR, M, and SS) is directly and positively linked to RIW, supporting hypotheses H2.1–H2.4. This aligns with the findings of Mitrović Veljković et al. (2020), who identified EI as the foundation for a more successful learning process. Similarly, J. Iqbal et al. (2022) reported that the relationship between EI and teacher–student interactions is naturally observed in university settings. Thus, the study concluded that higher EI is associated with greater metacognitive effort regulation in nursing education. This finding is consistent with that of Huang (2021), who reported that RIW is positively associated with student engagement.

Furthermore, the study measured the direct connection between RIW and MER, highlighting that RIW is directly and positively connected with MER, thus supporting Hypothesis H3. This finding is consistent with the outcomes reported by J. Iqbal et al. (2022). However, teacher support is significantly associated with the cognitive process of learning among higher education students. These studies, along with (Rickert & Skinner, 2022), provide contextual support for the idea that RIW is positively associated with learning processes, aligning well with the concept of higher metacognitive effort regulation.

The study also examined how RIW mediated the relationship between EI (SA, SR, M, and SS) and MER. The results confirmed that RIW may mediate the associations between all components of EI and MER, supporting H4.1–H4.4. These findings align with research demonstrating that EI, in combination with teacher relationships with students, is associated with cognitive function among higher education students in China (J. Iqbal et al., 2022). Additionally, the results corroborate the work of J. Iqbal et al. (2021a) and Prafitriyani et al. (2019), who reported that a link between EI and teacher–student engagement is associated with cognitive processes among students at the participating Pakistani institution. Overall, the study emphasized that RIW may be a mediator of the relationship between EI and MER. However, given the exploratory nature of the study and the newly developed MER measure, these findings should be treated as preliminary and hypothesis-generating.

## 10. Cross-Institutional Comparison via Multigroup Analysis

An important contribution of this study is the comparison between nursing students at the two participating institutions, using Henseler et al. (2016b) PLS-MGA with 5000 bootstrap samples and one-tailed significance testing (see Table 5 and Table 6). As reported in Section 8.2, the only significant difference between the two participating institutions was the path from self-regulation to relational instructor warmth, which was stronger among students at the participating Pakistani institution than at the participating Chinese institution. This suggests that students at the participating Pakistani institution may show a stronger association between their self-regulation abilities and perceived instructor warmth compared to students at the participating Chinese institution, while the overall theoretical model operates similarly across both contexts.

## 11. Conclusions

This exploratory study, based on prior literature and Goleman’s EI theory, explored the correlational relationships between EI and MER among nursing students at the two participating institutions in Pakistan and China. The results revealed that all four EI dimensions (SA, SR, M, and SS) were significantly and positively associated with MER. The study also examined EI’s direct correlational relationships with RIW, finding that SA, SR, M, and SS were positively and significantly associated with RIW. RIW was significantly correlated with MER and functioned as a partial mediator in the relationships between each EI dimension and MER at both participating institutions. This study led to several key insights into the correlational relationships between EI and MER among nursing students at the two participating institutions in Pakistan and China. First, SA, SR, M, and SS were each significantly associated with MER, highlighting their potential association with higher metacognitive effort regulation. Moreover, RIW positively correlated with all facets of EI, including SA, SR, M, and SS, among nursing students. A higher level of RIW was associated with greater MER in nursing educational institutions at both participating institutions. Finally, the findings suggest that a combination of SA, SR, M, and SS, supported by effective RIW, is associated with higher MER among nursing students. These findings suggest that EI and RIW may be associated with greater metacognitive effort regulation, though causal claims cannot be made. As an exploratory study using a newly developed MER measure, the primary contribution of this research is to generate hypotheses and inform future confirmatory research rather than to provide definitive evidence of causal relationships. Future research should validate the MER scale with independent samples before the findings can be treated as confirmatory.

## 12. Implications

The following suggestions are tentative, informed by the correlational findings, and intended to guide future research. They were not tested in this study and require experimental validation before implementation.

For future research. Researchers should test whether EI screening followed by targeted interventions (e.g., emotion labeling, cognitive reappraisal) improves MER outcomes. Experimental or quasi-experimental designs with control groups are needed. Researchers should also test whether instructor warmth training (e.g., empathy training, feedback timing) improves student MER outcomes.

For practitioners. If future research supports these associations, teachers might consider integrating brief EI-related activities into existing courses. However, no specific dosage, timing, or format can be recommended based on the present correlational findings.

For policymakers. Policymakers should fund longitudinal and experimental studies to establish causal ordering before implementing large-scale EI or warmth-based interventions. The present findings provide preliminary correlational evidence that may justify such investments.

## 13. Limitations and Future Research

As an exploratory study, this research has several limitations that affect the interpretation of findings. First, we employed convenience sampling from a single nursing college in each country, which introduces potential selection bias and severely limits the generalizability of our cross-institutional findings. The observed MGA difference (SR to RIW) may reflect institutional or regional characteristics rather than national culture, and future multi-institution, randomly sampled studies are needed to validate these findings. Second, our self-report measures of positive constructs (EI, RIW, MER) are susceptible to social desirability bias, potentially inflating correlations among focal constructs. Although we employed procedural remedies (anonymity, confidentiality assurances, Harman’s single-factor test explaining 28.61% of variance), future research should use multi-source (e.g., instructor ratings) or behavioral measures. Third, the study focuses on nursing education in Pakistan and China only, limiting broader generalizability. Fourth, we collected data from undergraduate nursing students only; other disciplines and graduate students were not included. Fifth, the cross-sectional design precludes causal inference; future studies should adopt longitudinal or time-lag designs to validate these outcomes. Future research could examine the association of EI and peer support with MER. Although theoretically grounded, the cross-sectional design cannot rule out alternative directions (e.g., RIW to EI or MER to RIW via EI); longitudinal or experimental designs are needed to confirm causal ordering. Sixth, formal content validity indices (I-CVI, S-CVI) were not computed. Although five experts reviewed the questionnaire and provided qualitative feedback that informed item revisions, future research should compute quantitative content validity indices to strengthen the instrument’s psychometric documentation. Seventh, and most importantly, the MER scale was newly developed for this exploratory study and requires independent validation. The conceptual overlap between MER and the self-regulation dimension of EI suggests that the two constructs may not be empirically distinct; future research should use confirmatory factor analysis and discriminant validity tests with independent samples to establish the measure’s validity. The exploratory nature of the study means that findings are hypothesis-generating rather than confirmatory, and all results related to MER should be interpreted with this limitation in mind. Finally, the study measured metacognitive effort regulation cross-sectionally. Future research should use longitudinal designs to examine how effort regulation develops and changes over time.

## Figures and Tables

**Table 1:** Reliability, validity and multicollinearity.

Variables	Items	Loading	VIF
Self-Awareness CA: 0.878 CR (rho_a): 0.883 CR (rho_c): 0.911 AVE: 0.673	SA1	0.838	2.078
	SA2	0.841	2.206
	SA3	0.825	2.042
	SA4	0.800	1.992
	SA5	0.796	1.853
Self-Regulation (SR) CA: 0.902 CR (rho_a): 0.909 CR (rho_c): 0.925 AVE: 0.672	SR1	0.860	2.586
	SR2	0.863	2.666
	SR3	0.844	2.338
	SR4	0.798	2.012
	SR5	0.777	1.881
	SR6	0.773	1.928
Motivation (M) CA: 0.863 CR (rho_a): 0.870 CR (rho_c): 0.907 AVE: 0.709	M1	0.850	2.185
	M2	0.876	2.503
	M3	0.851	1.972
	M4	0.789	1.747
Social Skills (SS) CA:0.915 CR (rho_a):0.921 CR (rho_c): 0.932 AVE:0.661	SS1	0.831	2.411
	SS2	0.855	2.647
	SS3	0.820	2.278
	SS4	0.793	2.002
	SS5	0.754	1.965
	SS6	0.794	2.102
	SS7	0.841	2.493
Relational instructor warmth (RIW) CA: 0.934 CR (rho_a): 0.936 CR (rho_c): 0.945 AVE: 0.655	RIW1	0.822	2.486
	RIW2	0.820	2.395
	RIW3	0.807	2.349
	RIW4	0.780	2.059
	RIW5	0.764	2.032
	RIW6	0.792	2.164
	RIW7	0.838	2.613
	RIW8	0.826	2.514
	RIW9	0.830	2.532
Metacognitive Effort Regulation (MER) CA: 0.885 CR (rho_a): 0.887 CR (rho_c): 0.916 AVE: 0.685	MER1	0.855	2.382
	MER2	0.848	2.329
	MER3	0.838	2.144
	MER4	0.806	1.941
	MER5	0.789	1.872

***Note.*** Cronbach’s alpha (CA), composite reliability (rho_a), composite reliability (rho_c), average variance extracted (AVE). EI was modeled as four separate dimensions (SA, SR, M, SS); each dimension was entered as a separate predictor in the structural model.

**Table 2:** Measuring the direct relationship between variables.

H	Direct Relationship	Coefficients	95% CI	Means	SD	T Value	*p* Values	Decision
H1.1	SA -> MER	0.192	[0.127, 0.257]	0.193	0.033	5.818	<0.001	Accepted
H1.2	SR -> MER	0.190	[0.119, 0.261]	0.189	0.036	5.269	<0.001	Accepted
H1.3	M -> MER	0.195	[0.124, 0.266]	0.195	0.036	5.435	<0.001	Accepted
H1.4	SS -> MER	0.223	[0.156, 0.290]	0.223	0.034	6.631	<0.001	Accepted
H2.1	SA -> RIW	0.106	[0.027, 0.185]	0.108	0.040	2.641	0.008	Accepted
H2.2	SR -> RIW	0.204	[0.126, 0.282]	0.205	0.040	5.161	<0.001	Accepted
H2.3	M -> RIW	0.146	[0.070, 0.222]	0.147	0.039	3.727	<0.001	Accepted
H2.4	SS -> RIW	0.171	[0.095, 0.247]	0.171	0.039	4.394	<0.001	Accepted
H3	RIW -> MER	0.217	[0.150, 0.284]	0.218	0.034	6.329	<0.001	Accepted
**Control** **variables**	Gender -> MER	0.051	[−0.076, 0.178]	0.050	0.065	0.789	0.430	Insignificant
	Study Years -> MER	0.027	[−0.038, 0.092]	0.027	0.033	0.809	0.419	Insignificant

***Note.*** SA = self-awareness; SR = self-regulation; M = motivation; SS = social skills; RIW = relational instructor warmth; MER = metacognitive effort regulation.

**Table 3:** Measuring the indirect relationship between variables.

H	Indirect Relationship	Coefficients	95% CI	Means	SD	T Value	*p* Values	Decision
H4.1	SA -> RIW -> MER	0.023	[0.005, 0.041]	0.023	0.009	2.432	0.015	Accepted
H4.2	SR -> RIW -> MER	0.044	[0.022, 0.066]	0.045	0.011	3.921	<0.001	Accepted
H4.3	M -> RIW -> MER	0.032	[0.012, 0.052]	0.032	0.010	3.268	0.001	Accepted
H4.4	SS -> RIW -> MER	0.037	[0.017, 0.057]	0.037	0.010	3.633	<0.001	Accepted

***Note.*** SA = self-awareness; SR = self-regulation; M = motivation; SS = social skills; RIW = relational instructor warmth; MER = metacognitive effort regulation.

**Table 4:** Total Effects of EI Dimensions, RIW, and Control Variables on MER.

H	Path	Coefficients	Means	SD	T Value	*p* Values	Decision
H1.1	SA → MER	0.215	0.216	0.034	6.383	<0.001	Significant
H1.2	SR → MER	0.234	0.234	0.036	6.514	<0.001	Significant
H1.3	M → MER	0.226	0.227	0.036	6.317	<0.001	Significant
H1.4	SS → MER	0.260	0.260	0.034	7.685	<0.001	Significant
H2.1	SA → RIW	0.106	0.108	0.040	2.641	0.008	Significant
H2.2	SR → RIW	0.204	0.205	0.040	5.161	<0.001	Significant
H2.3	M → RIW	0.146	0.147	0.039	3.727	<0.001	Significant
H2.4	SS → RIW	0.171	0.171	0.039	4.394	<0.001	Significant
H3	RIW → MER	0.217	0.218	0.034	6.329	<0.001	Significant
Control Variables	Gender → MER	0.051	0.050	0.065	0.789	0.430	Insignificant
	Study Years → MER	0.027	0.027	0.033	0.809	0.419	Insignificant

***Note.*** SA = self-awareness; SR = self-regulation; M = motivation; SS = social skills; RIW = relational instructor warmth; MER = metacognitive effort regulation.

**Table 5:** MICOM Results for Measurement Invariance Across Country Groups.

Construct	Step 2 (Compositional)	Step 3a (Means)	Step 3b (Variances)			
	Original r	*p*	Original Diff	*p*	Original Diff	*p*
Metacognitive effort regulation	1.000	0.796	0.029	0.699	0.099	0.323
Motivation	0.998	0.132	0.011	0.896	−0.281	0.002 *
Relational Instructor Warmth	1.000	0.511	−0.049	0.556	0.038	0.699
Self-Awareness	0.997	0.156	0.151	0.080	−0.236	0.014 *
Self-Regulation	0.999	0.497	−0.078	0.369	0.019	0.836
Social Skills	0.999	0.458	0.003	0.979	−0.062	0.509

Note. Step 2 confirms compositional invariance when *p* > 0.05. Step 3a confirms equal means when *p* > 0.05. Step 3b confirms equal variances when *p* > 0.05. Group 1 = Pakistan; Group 2 = China. * *p* < 0.05, indicating a significant difference.

**Table 6:** Multigroup Analysis (PLS-MGA) Results for Path Coefficients and Indirect Effects.

Relationship	Pakistan (β)	China (β)	Difference	*p* (1-Tailed)
Direct paths				
Gender → MER	0.090	0.028	0.067	0.625
Motivation → MER	0.247	0.174	0.074	0.343
Motivation → RIW	0.105	0.165	−0.060	0.487
RIW → MER	0.184	0.240	−0.059	0.448
Self-Awareness → MER	0.155	0.203	−0.047	0.498
Self-Awareness → RIW	0.078	0.135	−0.060	0.502
Self-Regulation → MER	0.188	0.199	−0.011	0.898
Self-Regulation → RIW	0.348	0.116	0.231	0.004 *
Social Skills → MER	0.188	0.234	−0.045	0.520
Social Skills → RIW	0.145	0.190	−0.045	0.587
Study Years → MER	−0.043	0.066	−0.109	0.120
Indirect paths (mediation)				
Self-Awareness → RIW → MER	0.014	0.032	−0.019	0.337
Self-Regulation → RIW → MER	0.084	0.028	0.036	0.176
Social Skills → RIW → MER	0.027	0.045	−0.019	0.347
Motivation → RIW → MER	0.019	0.040	−0.020	0.290

***Note.*** Group 1 = Pakistan; Group 2 = China. Difference = Pakistan—China. Henseler’s PLS-MGA with 5000 bootstrap samples was used (Henseler et al., 2016a). One-tailed *p*-values are reported, consistent with the PLS-MGA procedure. * *p* < 0.05, indicating a significant difference.

## Data Availability

The datasets presented in this article are not readily available because participants were assured of confidentiality and that their responses would be used solely for research purposes. The ethical approval granted for this study did not include provisions for public data deposition. Requests to access the datasets should be directed to the corresponding author, Muhammad Zaheer Asghar, and will be reviewed to ensure compliance with ethical commitments and participant confidentiality agreements.

## Appendix A. Questionnaire

**Self-Awareness**

SA1: I can recognize my emotions when faced with academic challenges.

SA2: I understand how my emotions influence my thoughts and behavior.

SA3: I am aware of how external factors affect my mood.

SA4: I am aware of my emotional strengths and weaknesses.

SA5: I can identify when my emotional state is changing.

**Self-Regulation**

SR1: I manage my emotions effectively when facing difficult learning tasks.

SR2: I can stop myself from overthinking when I need to focus on a task.

SR3: I stay calm and composed when I face unexpected academic difficulties.

SR4: I engage in positive activities to manage my mood when I feel down.

SR5: I can adjust my emotional reactions when a learning task becomes frustrating.

SR6: I avoid making impulsive decisions when I am upset about my academic performance.

**Motivation**

M1: I take responsibility for my emotional reactions to academic challenges.

M2: I remain determined in my academic goals despite obstacles.

M3: Learning from setbacks helps me improve my approach to studying.

M4: I manage my reactions in frustrating academic situations by staying focused on finding solutions.

**Social Skills**

SS1: I can adapt my communication style to suit different people in academic settings.

SS2: I enjoy encouraging others to share their ideas.

SS3: I find it easy to make friends in my academic environment.

SS4: I am comfortable collaborating with peers on group activities.

SS5: I can express my ideas clearly when working in group learning activities.

SS6: I find it easy to start conversations with new people in academic settings.

SS7: I can provide constructive feedback to my peers during group work.

**Relational Instructor Warmth (RIW)**

RIW1: My teacher shows genuine interest in my emotional well-being.

RIW2: My teacher responds with empathy when I share my academic struggles.

RIW3: My teacher uses positive reinforcement to acknowledge my efforts.

RIW4: I receive personalized feedback from my teacher that is supportive.

RIW5: My teacher creates a classroom atmosphere where mistakes are seen as learning opportunities.

RIW6: My teacher takes time to understand my personal learning challenges.

RIW7: I feel that my teacher genuinely cares about my academic success.

RIW8: My teacher creates a safe and respectful classroom environment.

RIW9: The supportive relationship I have with my teacher is important to me.

**Metacognitive Effort Regulation** (newly developed for this exploratory study; requires independent validation)

MER1: I use effortful cognitive strategies when completing academic tasks.

MER2: I persist with difficult academic tasks even when they require sustained mental effort.

MER3: I choose challenging learning tasks over easy ones that require less thought.

MER4: I resist the temptation to take mental shortcuts when learning complex material.

MER5: I invest mental effort in understanding material deeply rather than memorizing superficially.

**Note.** This five-item scale was newly developed for this study. While pilot testing and expert review provided preliminary evidence of reliability and content validity, the scale requires independent validation before findings can be treated as confirmatory.

**Informed Consent Form**

**Study Title**

Emotional Intelligence, Instructor Warmth, and Metacognitive Effort Regulation in Nursing Students: An Exploratory Two-Institution Correlational Study in China and Pakistan

**Introduction**

You are invited to participate in a research study conducted by researchers from Huazhong University of Science and Technology, Zhejiang International Studies University, and the University of Helsinki. Before deciding whether to participate, please carefully read the following information about the study.

**Purpose of the Study**

The purpose of this study is to explore how emotional intelligence relates to students’ learning behaviors, particularly metacognitive effort regulation, and to examine whether relational instructor warmth is associated with this relationship among nursing students from China and Pakistan.

**Procedures**

If you agree to participate, you will be asked to complete a questionnaire containing statements related to emotional intelligence, instructor–student relationships, and learning behaviors.

The questionnaire includes approximately 36 statements and will take about 10–15 min to complete. Your responses should reflect your personal experiences and opinions.

**Voluntary Participation**

Your participation in this study is completely voluntary. You may refuse to participate or withdraw from the study at any time without any penalty or negative consequences.

Choosing not to participate will not affect your academic status, relationship with your instructors, or access to educational services.

**Possible Risks and Benefits**

This study involves minimal risk. Some questions may require reflection on your learning experiences, emotions, or interactions with instructors. You may skip any question that makes you uncomfortable.

Although you may not receive a direct personal benefit, your participation may contribute to a better understanding of psychological and educational factors affecting nursing students’ learning experiences.

**Confidentiality and Privacy**

All information collected from participants will be treated confidentially. Your personal identity will not be disclosed in any reports, publications, or presentations resulting from this research.

The questionnaire does not require personally identifying information. Data will be analyzed only in aggregated form and will be used exclusively for academic research purposes.

**Data Storage and Use**

The collected data will be securely stored and accessed only by the research team. The information obtained from this study may be used for scientific publications and academic presentations while maintaining participant confidentiality.

**Ethical Approval**

This research study was approved by the Institutional Ethical Review Board of the School of Education, Huazhong University of Science and Technology (Approval No. IERB-SED/2305/2023, approved 23 May 2023) and by the Institutional Research Ethics Committee of the University of Education, Pakistan (Approval No. IREC/2023/10, approved 10 January 2023). The study was conducted in accordance with the principles of the Declaration of Helsinki.

**Contact Information**

**Principal Investigator:** Javed Iqbal, School of Artificial Intelligence for Humanities, Zhejiang International Studies University, Hangzhou, China. Email: javaid13688@hotmail.com

**Corresponding Researcher:** Muhammad Zaheer Asghar, Department of Education, University of Helsinki, Finland. Email: zaheer.asghar@helsinki.fi

**Participant Consent Statement**

By completing this questionnaire, I confirm that:

☐ I have read and understood the information provided above.

☐ I understand that my participation is voluntary.

☐ I understand that I may withdraw from participation at any time without consequences.

☐ I understand that my responses will remain confidential and will only be used for academic research purposes.

☐ I voluntarily agree to participate in this study.

Participant Name (optional): ___________________________

Participant Signature (optional): ________________________

Date: ___________________

Researcher Signature: _________________________________

Date: ___________________
